# Neighborhood Greenness Attenuates the Adverse Effect of PM_2.5_ on Cardiovascular Mortality in Neighborhoods of Lower Socioeconomic Status

**DOI:** 10.3390/ijerph16050814

**Published:** 2019-03-06

**Authors:** Maayan Yitshak-Sade, Peter James, Itai Kloog, Jaime E. Hart, Joel D. Schwartz, Francine Laden, Kevin J. Lane, M. Patricia Fabian, Kelvin C. Fong, Antonella Zanobetti

**Affiliations:** 1 Exposure, Epidemiology, and Risk Program, Department of Environmental Health, Harvard T.H. Chan School of Public Health, Boston, MA 02215, USA; rejch@channing.harvard.edu (J.E.H.); jschwrtz@hsph.harvard.edu (J.D.S.); francine.laden@channing.harvard.edu (F.L.); kcf502@mail.harvard.edu (K.C.F.); azanobet@hsph.harvard.edu (A.Z.); 2 Department of Population Medicine, Harvard Medical School and Harvard Pilgrim Health Care Institute, Boston, MA 02215, USA; pjames@hsph.harvard.edu; 3 Department of Geography and Environmental Development, Faculty of Humanities and Social Sciences, Ben-Gurion University, Beer-Sheva 8410501, Israel; ikloog@bgu.ac.il; 4 Channing Division of Network Medicine, Department of Medicine, Brigham and Women’s Hospital and Harvard Medical School, Boston, MA 02215, USA; 5 Department of Environmental Health, Boston University School of Public Health, Boston, MA 02118, USA; klane@bu.edu (K.J.L.); pfabian@bu.edu (M.P.F.)

**Keywords:** PM_2.5_, neighborhood greenness, modification, walkability

## Abstract

Features of the environment may modify the effect of particulate matter ≤2.5 µm in aerodynamic diameter (PM_2.5_) on health. Therefore, we investigated how neighborhood sociodemographic and land-use characteristics may modify the association between PM_2.5_ and cardiovascular mortality. We obtained residence-level geocoded cardiovascular mortality cases from the Massachusetts Department of Public Health (*n* = 179,986), and PM_2.5_ predictions from a satellite-based model (2001–2011). We appended census block group-level information on sociodemographic factors and walkability, and calculated neighborhood greenness within a 250 m buffer surrounding each residence. We found a 2.54% (1.34%; 3.74%) increase in cardiovascular mortality associated with a 10 µg/m^3^ increase in two-day average PM_2.5_. Walkability or greenness did not modify the association. However, when stratifying by neighborhood sociodemographic characteristics, smaller PM_2.5_ effects were observed in greener areas only among cases who resided in neighborhoods with a higher population density and lower percentages of white residents or residents with a high school diploma. In conclusion, the PM_2.5_ effects on cardiovascular mortality were attenuated by higher greenness only in areas with sociodemographic features that are highly correlated with lower socioeconomic status. Previous evidence suggests health benefits linked to neighborhood greenness may be stronger among lower socioeconomic groups. Attenuation of the PM_2.5_–mortality relationship due to greenness may explain some of this evidence.

## 1. Introduction

Cardiovascular disease (CVD) is the most common cause of death in the United States [1]. In the late 20th century there was a declining trend in CVD mortality attributable to health care and public health advancements, but CVD mortality is no longer improving [2].

Numerous clinical, behavioral, and personal characteristics, such as age, sex, obesity, and smoking, have been established as risk factors for the development of CVD [3]. Yet, beyond these well-known clinical factors, there are a wide range of environmental and sociodemographic exposures that might influence CVD risk [4] including air pollution [5,6,7,8,9,10], socioeconomic status (SES) [11,12], and neighborhood greenness [13].

Air pollution, a well-established risk factor for CVD morbidity and mortality [5,6,7,8,9,10], was identified recently by the Global Burden of Disease study as a highly ranked risk factor for CVD death [14]. A recent meta-analysis concluded that the relative risk for myocardial infarction is 1.02 times higher for every 10 µg/m^3^ increment increase in short-term exposure to PM_2.5_ [15]. Another systematic review that pooled studies of short-term exposure to PM_2.5_ found a 0.80% increase in CVD mortality per 10 µg/m^3^ increment increase in exposure [16].

Both traditional CVD risk factors and air pollution exposures are related to neighborhood sociodemographic and land-use characteristics. There is an increasing prevalence of some traditional CVD risk factors (such as diabetes, obesity, and hypertension) over the last two decades and there is evidence that people of lower socioeconomic status are at higher risk [11,12]. This link between low socioeconomic status and CVD risk may be related to residence in areas with high levels of air pollution combined with little financial and physical access to health care services [17,18,19,20]. Sociodemographic characteristics were found to modify the association between PM_2.5_ and mortality in several studies, showing larger effects among communities of non-white race [21], lower socioeconomic status [21,22], lower education [23], and higher unemployment rate [24,25].

Other neighborhood land-use characteristics such as neighborhood greenness and walkability may affect human health directly or through modification of the PM_2.5_ effect on health. A number of studies have defined neighborhood greenness as the amount of vegetation in a defined buffer around the residential address [13,26,27]. The common hypothesized mechanisms used to explain the beneficial effect of green space on cardiovascular health involve providing a location for physical activity [28], increasing social engagement, and lowering stress levels [29,30]. Greenness is also thought to reduce exposure to air pollution either by filtering the air or, more likely, by creating a buffer space between sources and individuals who might be exposed [31]. Neighborhood walkability is a concept of how amenable an area is for routine walking. Walkability indices are commonly comprised of land-use components that are linked to walking behavior [32] and measures of the neighborhood’s accessibility, connectivity and density [26,27]. A highly walkable built environment may be correlated with higher air pollution levels, and may lead to higher personal exposure to air pollution as individuals are spending more time outdoors [27]. At the same time, it may be more conducive to physical activity.

While studies have assessed the direct effect of these land-use characteristics on human health [13,27,33], evidence on whether they modify PM_2.5_ effects is scarce. In recent years, the few studies that assessed the modification of the PM_2.5_ effect by neighborhood greenness have shown contradicting results. For example, one study found higher air quality associated health risks among people who reported not using green areas [34]. Another study found larger mortality effect estimates with increasing neighborhood greenness [35]. One study has shown conflicting effect modification of the association between air pollution and mortality by neighborhood greenness across urban and rural areas [36]. To our knowledge, only one study has examined the modification of the PM_2.5_ effect on mortality risk by neighborhood walkability, and has found no evidence of an interaction between PM_2.5_ and walkability [37].

In this study, we aim to assess the joint modification of the PM_2.5_ related cardiovascular risk by land-use characteristics (neighborhood greenness and walkability) and neighborhood sociodemographic characteristics (income, race, population density, and education) while controlling for individual sociodemographic characteristics. 

## 2. Materials and Methods

### 2.1. Data and Study Population

The Massachusetts Department of Public Health registers mortality records with information on residential address, place of death, age at death, gender, ethnicity, education, occupation, the exact date of death, and the underlying cause of death. We obtained the records of all decedents who died of a cardiovascular cause (International Classification of Disease, 10th Revision, group I) [38] between the years 2001–2011 and were 40 years or older.

This study was approved by the Harvard T.H. Chan School of Public Health Human Subjects Committee and by the Massachusetts Department of Public Health. 

### 2.2. Air Pollution Exposure

We estimated average daily PM_2.5_ from a model generating predictions at a 1 × 1 km spatial resolution. Briefly, mean daily PM_2.5_ concentrations were estimated daily in each 1 × 1 km grid cell by calibrating Aerosol Optical Depth (AOD) with monitored PM_2.5_ using mixed effect models with spatial and temporal predictors and a random slope for day and nested regions. Out-of-sample “ten-fold” cross-validation showed excellent model performance (mean out-of-sample R^2^  =  0.88). For a more in-depth description, please refer to Kloog et al. (2014) [39]. We assigned each decedent the daily values for the grid in which his/her residential address was located.

### 2.3. Confounders

We adjusted our models for temperature and day of the week. We estimated daily average temperature from a model that incorporated moderate resolution imaging spectroradiometer (MODIS) land surface temperatures (LST) data, land-use regression (LUR) variables (percent urban, elevation, normalized difference vegetation index) and a random intercept and slope for surface temperature for each day. Out-of-sample ten-fold cross-validation showed excellent model performance (mean out-of-sample R^2^ = 0.94). For more in-depth information, refer to Kloog et al. (2014) [40]. As with PM_2.5_, each decedent was assigned the daily values for the grid in which his/her residential address was located.

### 2.4. Modifiers

We considered neighborhood land-use characteristics and sociodemographic characteristics as potential modifiers.

#### 2.4.1. Land-Use Modifiers

Neighborhood greenness was estimated using a 250 × 250 m spatial grid, and each decedent was assigned the value for the grid in which his/her address was located using the Normalized Difference Vegetation Index (NDVI). Seasonal NDVI estimates were obtained from MODIS from NASA’s Terra satellite for all the years included in the study period. NDVI exposure in the season of death was assigned to each subject. During the photosynthesis process, chlorophyll in plants strongly absorb visible light (0.4–0.7 μm) while leaves reflect near-infrared light (0.7–1.1 μm). The satellite image provides information on these two measurements, and the NDVI calculates the ratio of the difference between the near-infrared region and red reflectance to the sum of these two measures. NDVI values range from −1.0 to 1.0, with larger values indicating higher levels of vegetative density. Previous studies of neighborhood greenness have used a 250 m area and a larger buffer size (1000–1250 m radius) as an approximate measure of residential and walking greenness exposure, respectively [41,42,43]. Therefore, as a sensitivity analysis, we used the seasonal mean NDVI data in a 1250 m area surrounding each address [27]. 

*Neighborhood walkability index* at the census block group-level was assigned based on the US Environmental Protection Agency (EPA) Smart Location Database. The Smart Location Database provides nationwide geographic data on 90 attributes summarizing characteristics such as housing density, diversity of land-use, neighborhood design, destination accessibility, transit service, employment, and demographics at the census block group-level [44]. We created a nationwide walkability index combining *z*-scores of three components: population density (people/acre) on unprotected land; street intersection density (weighted, with auto-oriented intersections eliminated); and land-use diversity based on the mix of retail, office, service, industrial, entertainment, education, healthcare, and public administration employment in the census block group. The index ranges between −2.9 and 16, where higher values indicate a more walkable neighborhood [44]. 

#### 2.4.2. Neighborhood-Level Sociodemographic Modifiers

We obtained the following characteristics for each block group from the 2014 Census 5-year estimates from the American Community Survey (ACS) which spans from 1 January 2010 to 31 December 2014: percentage of residents by race (white, black or other), percent of residents with no high school diploma, median household income, percent of residents below the poverty line, and population density. 

### 2.5. Statistical Analysis

We assessed the association between CVD mortality and PM_2.5_ using the time-stratified case-crossover approach [45], with the case day defined as the date of death for each person. Control days were defined as every 3rd day before and after the case day, and within the same month and year as the case day. Based on our previous work, we defined the exposure window for the main analysis as the moving average concentration of PM_2.5_ of the current and previous days. All models were adjusted for day of the week and temperature [40], with the same exposure window as PM_2.5_. Personal SES is controlled for by design as the same person is compared on case and control days. Season is controlled for by design as cases and controls are in the same month.

We assessed the modification of the PM_2.5_ and CVD mortality association in three phases. First, we assessed modification by neighborhood greenness and walkability independently by separately adding multiplicative interaction terms for each. Then, we assessed joint modification by adding interaction terms between PM_2.5_; each land-use characteristic, and each of the neighborhood sociodemographic characteristics. Then, for simplicity, we presented the modification of the PM_2.5_ effects by the land-use characteristics in models stratified by the neighborhood sociodemographic characteristics.

To make sure the modifying effect of NDVI is not influenced by seasonality, we added a sensitivity analysis in which we assessed the modification effect of the PM_2.5_–CVD association by annual averages of NDVI. 

Results are presented for each 10 µg/m^3^ increase in PM_2.5_ at the 25^th^ and 75^th^ percentiles of the land-use modifier within each stratum (below or above the median value) of neighborhood sociodemographic characteristic. Analyses were performed using the ‘survival’ package in R version 3.5.2 [46].

## 3. Results

We included 179,986 persons who resided in Massachusetts and died of CVD between 2001 and 2011. The mean age of death was 80 years, about 94% of the decedents were white, 45% were males, and the majority had a high school education or less (Table 1). The interquartile range (IQR) of the two-day average concentration of PM_2.5_ was between 6.8 µg/m^3^ and 12.3 µg/m^3^, and the mean value was 10.2 µg/m^3^. Over the study period, the mean block group PM_2.5_ values were lower in rural areas and higher in urban areas, as expected, reaching a maximum value of 17.4 µg/m^3^ (Figure S1). The mean NDVI ranged between 0.00 and 0.87, and the mean walkability index ranged between −2.93 and 16.87. As expected, the walkability index was highest in urban areas and lowest in rural areas (Figure S2). NDVI tended to be higher in rural areas and lower in urban areas, although this was not true in all locations (Figure S3).

An increase of 10 µg/m^3^ in exposure to PM_2.5_ in lag days 0–1 was statistically significantly associated with a 2.54% (1.34%; 3.74%) increase in CVD mortality. No association was observed with exposure to temperature in lag days 0–1 (−1.38% (−3.49%; 0.77%), per 10 °C).

### 3.1. Modification by Land-Use Characteristics

Among all available cases, NDVI and walkability did not modify the association with PM_2.5_. The PM_2.5_–mortality association did not change across levels of NDVI. Results were similar when calculating the NDVI value in the 250 m and 1250 m buffer around the decedents’ addresses (Table 2).

### 3.2. Joint Modification by Land-Use and Neighborhood Sociodemographic Characteristics

When we assessed the joint modification effect of NDVI or walkability and the neighborhood sociodemographic characteristics, we observed a statistically significant joint modification of the PM_2.5_–CVD associations by NDVI and population density (interaction *p* value = 0.001), NDVI and percentage of white population (interaction *p* value = 0.019), and NDVI and percentage of persons with no high school diploma (interaction *p* value = 0.043). We found no joint modification by walkability and the neighborhood sociodemographic characteristics (Table S1). 

**Figure 1:**
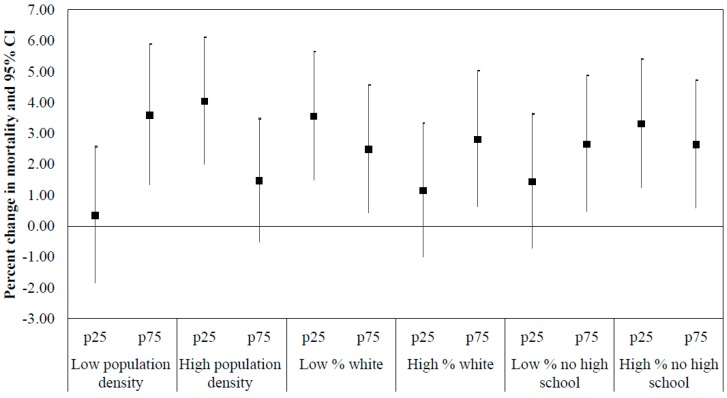
The percent increase and 95% confidence intervals in cardiovascular mortality, associated with PM_2.5_, in the 25^th^ and 75^th^ percentiles of NDVI, by neighborhood sociodemographic characteristics. p25 and p75 refer to percentiles of NDVI.

We observed smaller PM_2.5_ effects with increasing values of NDVI among people who resided in areas of higher population density, areas with a lower percentage of white population, and areas with a higher percentage of residents without a high school diploma. In areas of lower population density, a higher percentage of white residents, or a lower percentage of residents without a high school diploma, we observed larger PM_2.5_ effects with increasing values of NDVI (Figure 1 and Table S2). The sensitivity analysis, using annual NDVI averages, showed similar results (Figure S4). We also observed smaller PM_2.5_ effects with increasing values of NDVI in neighborhoods with a higher percentage of poverty and lower median household income, though these findings were not statistically significant (data not shown).

## 4. Discussion

In this study, we observed statistically significant increased risk for CVD mortality associated with exposure to PM_2.5_. Among all cases, the risk was not modified by neighborhood greenness or walkability. However, we found that PM_2.5_-related cardiovascular mortality was lower with higher NDVI only in neighborhoods with higher population density, a lower percentage of white population, or a higher percentage of residents without a high school diploma. Although the joint interaction was not statistically significant, we observed smaller PM_2.5_ effects in greener neighborhoods with a higher percentage of poverty and lower median household income as well. No joint effect modification was observed for walkability.

Our finding of joint effect modification of the PM_2.5_–CVD mortality association, by NDVI and sociodemographic factors, is generally consistent with evidence from previous studies suggesting that the health benefits of exposure to green space are stronger among low socioeconomic groups and in urban areas [36,47,48]. Similar to our findings, a study that assessed the association between green space and cardiovascular mortality found reduced mortality risk among those exposed to greener environments only amongst the most socioeconomically deprived groups [48]. A study that included 49 U.S. cities found protective effects of neighborhood greenness on mortality and life expectancy only in areas of lower socioeconomic status [28]. Another study conducted in the U.S. found a positive association between access to quality green space and reduced psychological distress among deprived urban populations [49]. A possible explanation for why the effect of air pollution is weaker in greener neighborhoods with lower socioeconomic status may be related to different usage of green space in these areas. Higher access to green space among deprived communities may contribute to a reduction of health inequalities by increasing frequency or time spent on outdoor activities, which contribute to a better psychological and physical health [50]. In addition, deprived communities may get a larger benefit from green spaces because residents of these communities may be more likely to spend time outside and interact with their immediate neighborhood compared to those in wealthier neighborhoods [51,52,53].

To our knowledge, there are no studies that assessed the difference in the joint modification of the PM_2.5_ effect by greenness and SES levels. However, studies that assessed the modification of the PM_2.5_ effect by SES alone found that poorer neighborhoods are often characterized by higher pollution levels and larger PM_2.5_ effects [20]. 

Another finding of our study is the synergistic effect of PM_2.5_ and greenness found in neighborhoods with a lower population density, higher percentage of whites, and higher education level. A possible explanation for this finding is that the benefits of neighborhood greenness could be compromised by conditions and lifestyles that are prevalent in greener rural areas (such as car dependency) and distance to health care [28]. 

We found no modification of the effect of PM_2.5_ by neighborhood walkability. Like our findings, a study in California that compared the ischemic heart disease mortality risk between neighborhoods according to their walkability score found that the estimated mortality risk contributed by air pollution exposure did not differ by the neighborhood walkability score [37].

A major strength of this study is the use of geocoded mortality cases with high spatially and temporally resolved exposure. In addition, using joint interaction analysis, we were able to assess the complex relationship between multiple environmental exposures and mortality.

Our study had several limitations. First, similarly to other studies that assess the effect of air pollution on human health, there is a possibility of exposure measurement error due to the lack of information on participants’ activity space. Second, we did not have the ability to evaluate NDVI and walkability at the same exposure window as PM_2.5_. NDVI was calculated seasonally, and walkability was calculated once during the study period. However, since these land-use characteristics vary slowly over time, we do not expect it to bias our results. Third, the sociodemographic neighborhood-level modifiers included in our analysis may not have adequately captured differences across neighborhoods and possibly underestimated the neighborhood effects. In addition, in this study design, we were not able to account for personal usage of green space.

## 5. Conclusions

In conclusion, PM_2.5_-related CVD mortality risk was smaller in highly populated greener neighborhoods with lower SES. Cumulative evidence suggests that health benefits linked to neighborhood greenness may be stronger among the lower socioeconomic groups. This may explain the beneficial effect of greenness found only in highly dense neighborhoods with a lower percentage of white residents and lower education. Our findings confirm the importance of addressing multiple aspects of an individual’s environment when assessing the effect of air pollution on human health.

## Figures and Tables

**Table 1:** Population characteristics: cardiovascular mortality cases in Massachusetts 2001–2011.

Population Characteristics	*N* = 179,986
Male gender, *n* (%)	81,857 (45.5)
Race, *n* (%)	
White	169,735 (94.3)
Black	6624 (3.7)
Other	3627 (2.0)
Education, *n* (%)	
Elementary	34,909 (19.7)
High school	94,283 (53.1)
some college	21,020 (11.8)
College or more	27,348 (15.4)
Place of death, *n* (%)	
Hospital	81,621 (45.3)
Out of the hospital	98,293 (54.6)
Unknown	72 ( 0.0)
Age, Mean (SD)	80.37 (12.3)

**Table 2:** The percent change in cardiovascular mortality associated with 10 µg/m^3^ increase in PM_2.5_ in the 25^th^ and 75^th^ percentiles of NDVI and walkability measures.

Modifier	Interaction *p* Value	Percent Change in Mortality (95% CI)	
		25^th^ Percentile of the Modifier	75^th^ Percentile of the Modifier
^1^ Walkability	0.165	2.03% (0.64%; 3.43%)	2.92% (1.52%; 4.33%)
^2^ NDVI (250 m buffer)	0.804	2.42% (0.93%; 3.94%)	2.65% (1.15%; 4.16%)
^2^ NDVI (1250 m buffer)	0.613	2.33% (0.81%; 3.88%)	2.81% (1.28%; 4.36%)

^1^ The walkability index was created using the z scores of the following three components of the US EPA Smart Location Database: (1) gross population density (i.e., people/acre on unprotected land); (2) eight-tier employment entropy (i.e., land-use diversity of employment mix of retail, office, service, industrial, entertainment, education, healthcare, and public administration occupations); (3) street intersection density (i.e., summary of the total intersection density, weighted to reflect connectivity for pedestrian and bicycle travel). ^2^ Normalized Difference Vegetation Index (NDVI) is the ratio of the difference between the near-infrared region and red reflectance and the sum of these two measures. NDVI values range from −1.0 to 1.0, with larger values indicating higher levels of vegetative density.

## Supplementary Materials

The following are available online at https://www.mdpi.com/1660-4601/16/5/814/s1, Table S1: The joint modification of the PM_2.5_ effect by NDVI, walkability and neighborhood sociodemographic characteristics, Table S2: Percent increase in mortality for 10 µg/m^3^ of average PM_2.5_ in the same and previous day: modification by NDVI within strata of low and high sociodemographic characteristic, Figure S1: Block group average concentrations of PM_2.5_ in the study area across Massachusetts (2001–2011), Figure S2: Block group average walkability index in the study area across Massachusetts (2001–2011), Figure S3: Block group average NDVI in the study area across Massachusetts (2001–2011), Figure S4: The percent increase and 95% confidence intervals in cardiovascular mortality, associated with PM_2.5_, in the 25th and 75th percentiles of NDVI, by neighborhood sociodemographic characteristics.
