# Supplementary material for: Neighborhood Greenness Attenuates the Adverse Effect of PM2.5 on Cardiovascular Mortality in Neighborhoods of Lower Socioeconomic Status
Source: Int J Environ Res Public Health. 2019 Mar 6;16(5):814. doi: 10.3390/ijerph16050814 (PMC6427452; doi:10.3390/ijerph16050814)
Supplement: Supplementary file 1 [file ijerph-16-00814-s001.pdf]

Supplemental Material: The Joint Modification of the $PM_{2.5}$ and Mortality Association by Neighborhood Land-Use and Sociodemographic Characteristics

Table S1. The joint modification of the $PM_{2.5}$ effect by NDVI, walkability and neighborhood sociodemographic characteristics.

Sociodemographic Characteristics	[1] $PM_{2.5}$:NDVI:SC β (*p* value)	[2] $PM_{2.5}$:walkability:SC β (*p* value)
Population density	-2.20 (0.001)	0.01 (0.838)
Percent 65 and older	0.03 (0.201)	0.01 (0.415)
Percent white	0.03 (0.019)	-0.01 (0.953)
Percent less than high school	-0.06 (0.043)	-0.01 (0.845)
Percent poverty	-0.11 (0.186)	-0.02 (0.173)
Median household income	0.01 (0.106)	-0.01 (0.857)

[1] The interaction p value of the joint interaction between $PM_{2.5}$, NDVI and each of the neighborhood sociodemographic characteristics (SC). [2] The interaction p value of the joint interaction between $PM_{2.5}$, walkability and each of the neighborhood sociodemographic characteristics (SC).

Table S2. Percent increase in mortality for 10μg/m^3 of average $PM_{2.5}$ in the same and previous day: modification by NDVI within strata of low and high SES.

SES strata	NDVI percentile	Percent	Lower CI	Higher CI
Low population density	25th	0.34%	-1.84%	2.56%
	75th	3.58%	1.33%	5.88%
High population density	25th	4.03%	2.00%	6.10%
	75th	1.46%	-0.52%	3.47%
Low % white	25th	3.55%	1.49%	5.65%
	75th	2.47%	0.43%	4.56%
High % white	25th	1.14%	-1.00%	3.33%
	75th	2.80%	0.62%	5.02%
Low % no high school diploma	25th	1.42%	-0.72%	3.62%
	75th	2.64%	0.46%	4.86%
High % no high school diploma	25th	3.31%	1.26%	5.41%
	75th	2.64%	0.60%	4.72%

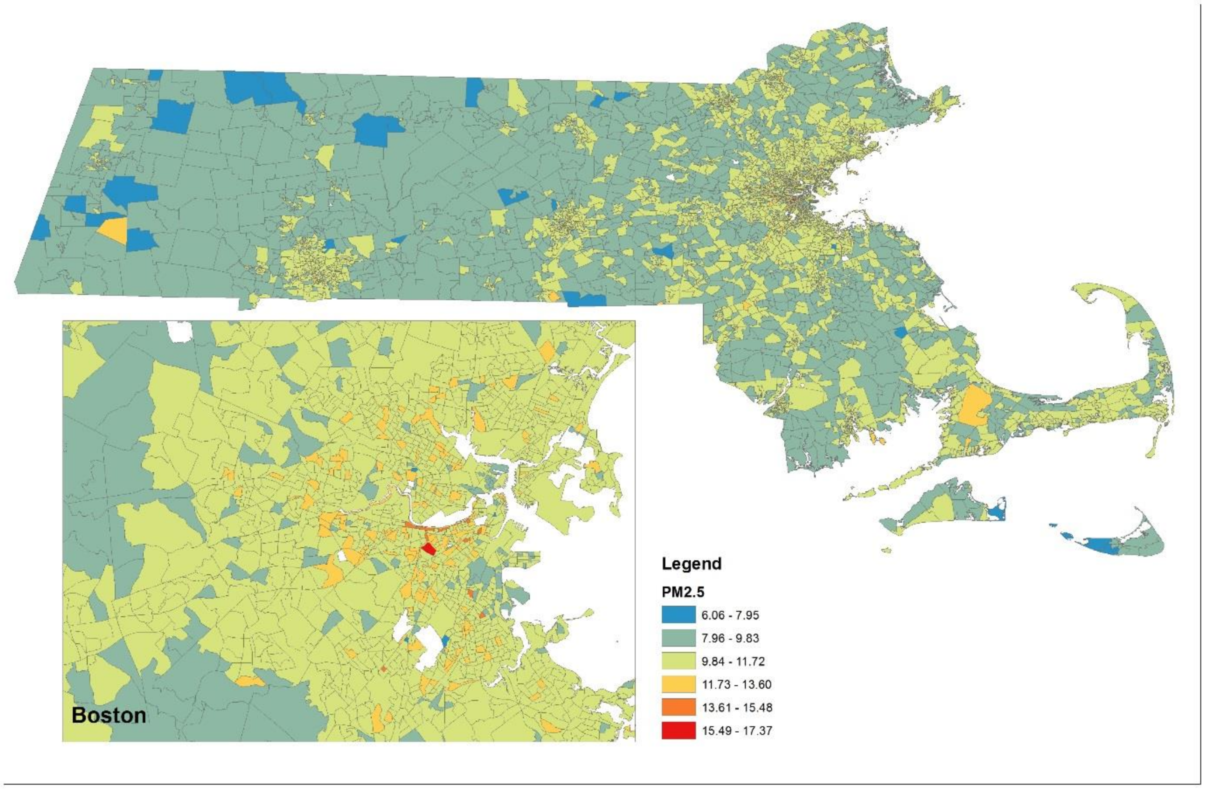

Figure S1. Block group average concentrations of $PM_{2.5}$ in the study area across Massachusetts (2001-2011).

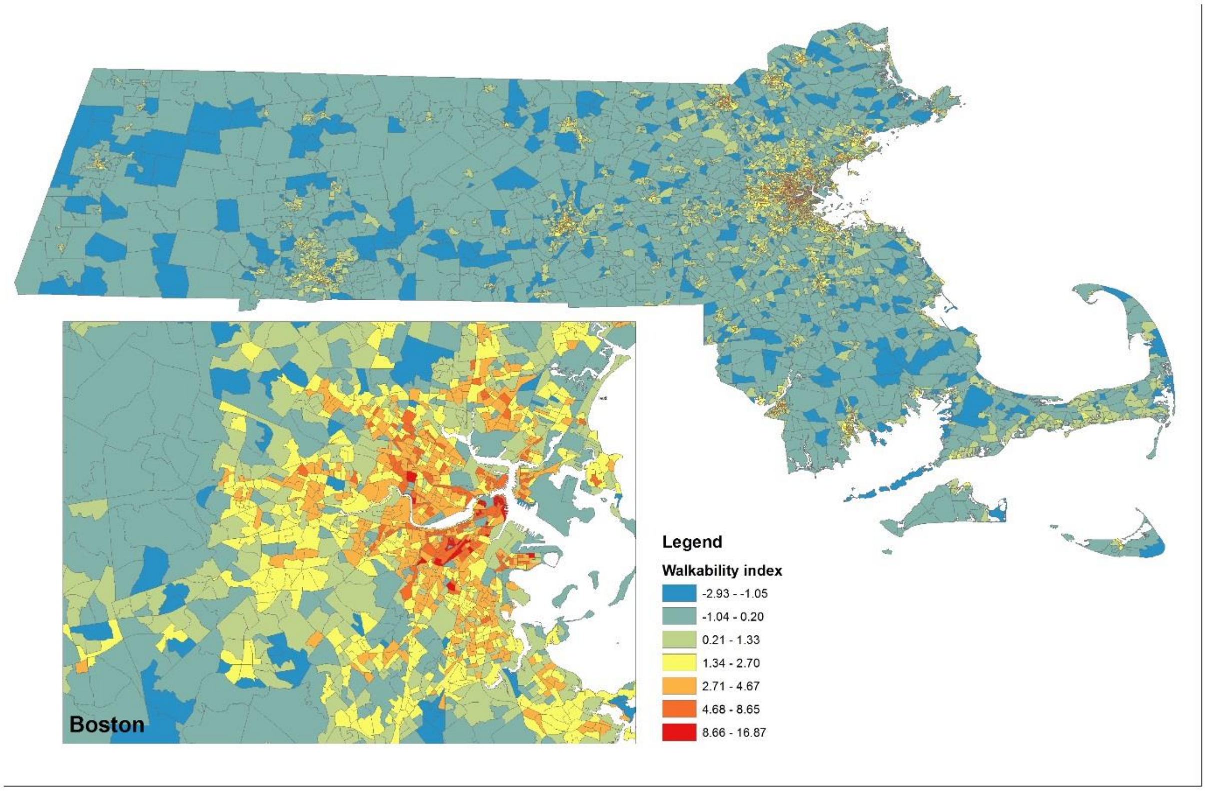

Figure S2. Block group average walkability index in the study area across Massachusetts (2001-2011).

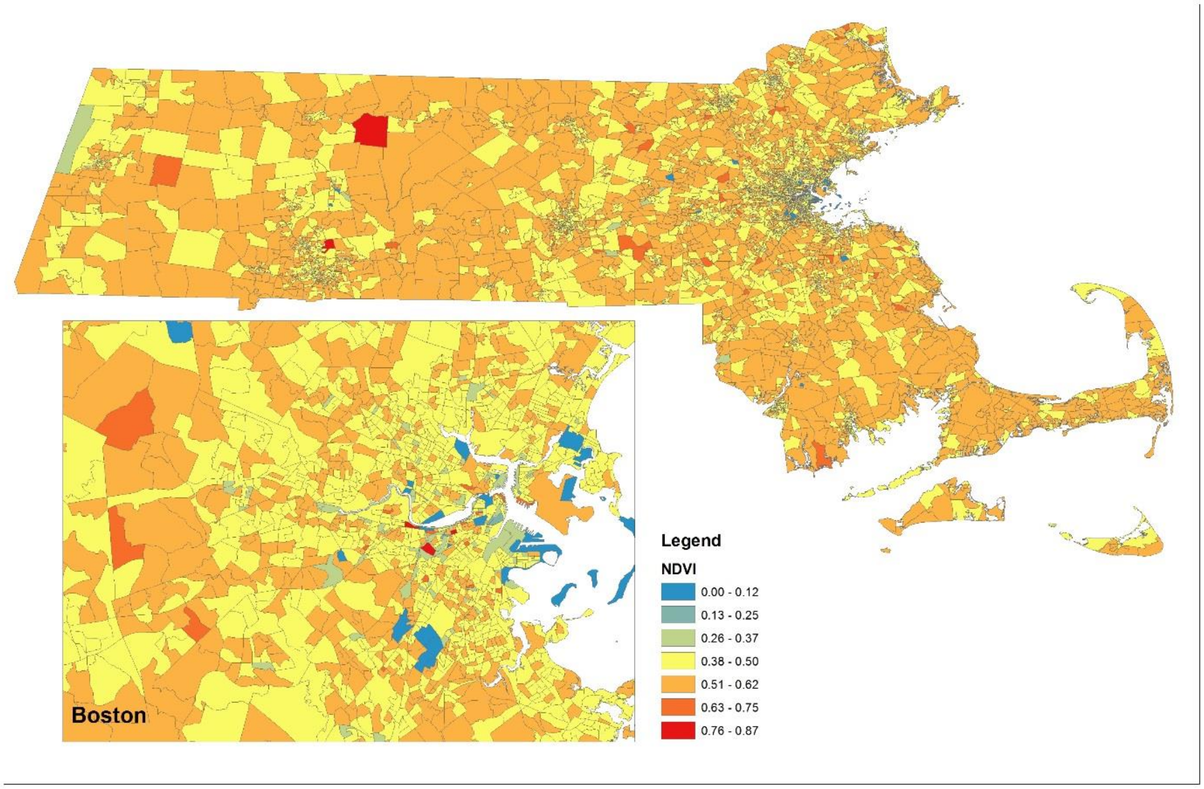

Figure S3. Block group average NDVI in the study area across Massachusetts (2001-2011).

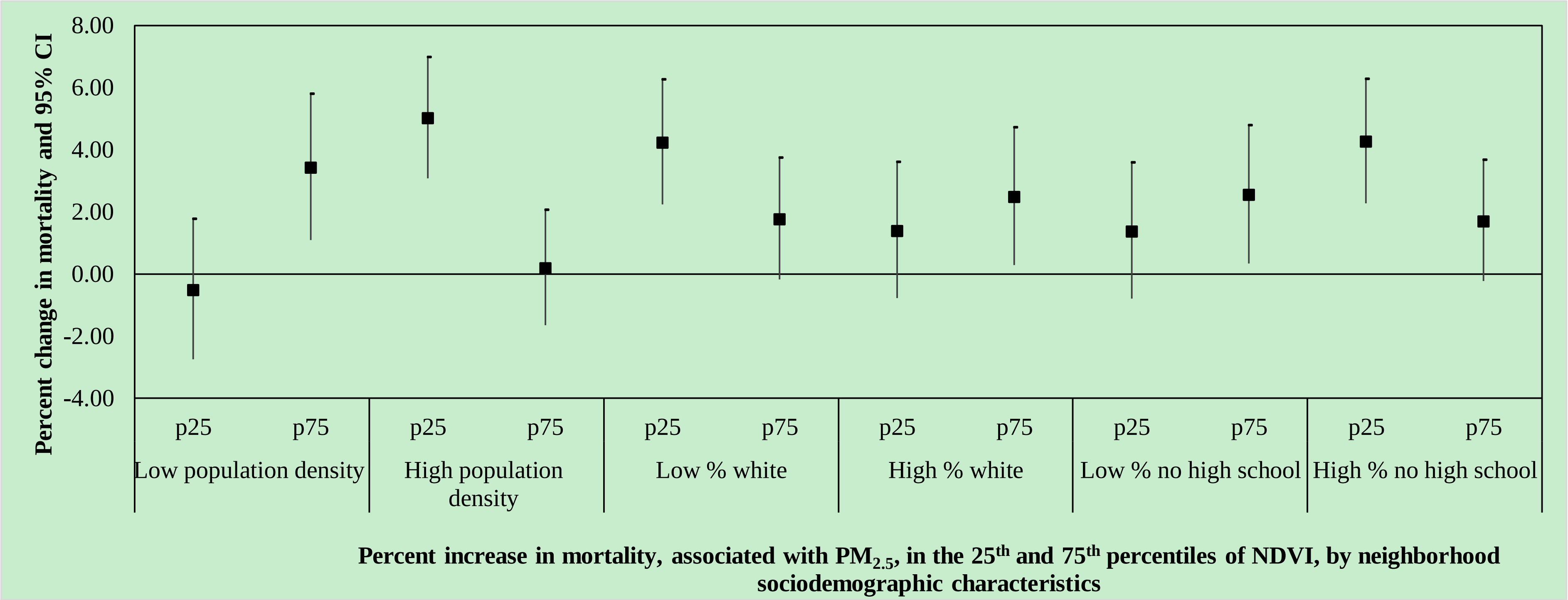

Figure S4. The Percent increase and 95% confidence intervals in cardiovascular mortality, associated with $PM_{2.5}$, in the 25th and 75th percentiles of NDVI, by neighborhood sociodemographic characteristics. P25 and p75 refer to percentiles of annual NDVI.
